# Stimson’s Operative Surgery

**Published:** 1901-03

**Authors:** 


### Books and Magazines.

---

STIMSON'S OPERATIVE SURGERY. A Manual of Operative Surgery, by Lewis A. Stimson, B. A., M. D., Professor of Surgery in Cornell University Medical College. New (4th) and thoroughly revised edition. In one royal 12mo. volume of 581 pages with 293 illustrations. Just ready. Cloth, $3.00, net. Lea Brothers & Co., Philadelphia and New York.

This valuable book has not been increased in size over its three former editions. but has been reduced. In the revision which it has been through much of the text and about forty of the cuts have been omitted. The present advancement of surgery has rendered many of the cuts obsolete. There are 293 illustrations, which cover every phase of operative surgery.

